# Variability in the timeliness of interventional radiology availability for angioembolization of hemodynamically unstable pelvic fractures: a prospective survey among U.S. level I trauma centers

**DOI:** 10.1186/s13037-019-0201-9

**Published:** 2019-06-20

**Authors:** Stephanie Jarvis, Alessandro Orlando, Benoit Blondeau, Kaysie Banton, Cassandra Reynolds, Gina M. Berg, Nimesh Patel, Michael Kelly, Matthew Carrick, David Bar-Or

**Affiliations:** 1 Trauma Research, LLC, 383 Corona St. #319, Denver, CO 80218 USA; 2 Research Medical Center, 2316 East Meyer Blvd, Kansas City, MO 64132 USA; 3 University of Connecticut, Hartford Hospital, Hartford, CT 06106 USA; 4 Swedish Medical Center, 501 E Hampden Ave, Englewood, CO 80113 USA; 5 Wesley Medical Center, 550 N. Hillside St, Wichita, KS 67214 USA; 6 St. Anthony’s Hospital, 11600 West 2nd Place, Lakewood, CO 80228 USA; 7 Penrose Hospital, 2222 North Nevada Ave, Colorado Springs, CO 80907 USA; 8 Medical City Plano, 3901 West 15th Street, Plano, TX 75075 USA

**Keywords:** Pelvic fracture management, Interventional radiology, Angioembolization, Resuscitative endovascular balloon occlusion of the aorta

## Abstract

**Background:**

Patients with hemodynamically unstable pelvic fractures have high mortality due to delayed hemorrhage control. We hypothesized that the availability of interventional radiology (IR) for angioembolization may vary in spite of the mandated coverage at US Level I trauma centers, and that the priority treatment sequence would depend on IR availability.

**Methods:**

This survey was designed to investigate IR availability and pelvic fracture management practices. Six email invitations were sent to 158 trauma medical directors at Level I trauma centers. Participants were allowed to skip questions and irrelevant questions were skipped; therefore, not all questions were answered by all participants. The primary outcome was the priority treatment sequence for hemodynamically unstable pelvic fractures. Predictor variables were arrival times for IR when working off-site and intervention preparation times. Kruskal-Wallis and ordinal logistic regression were used; alpha = 0.05.

**Results:**

Forty of the 158 trauma medical directors responded to the survey (response rate: 25.3%). Roughly half of participants had 24-h on-site IR coverage, 24% (4/17) of participants reported an arrival time ≥ 31 min when IR was on-call. 46% (17/37) of participants reported an IR procedure setup time of 31–120 min. Arrival time when IR was working off-site, and intervention preparation time did not significantly affect the sequence priority of angioembolization for hemodynamically unstable pelvic fractures.

**Conclusions:**

Trauma medical directors should review literature and guidelines on time to angioembolization, their arrival times for IR, and their procedural setup times for angioembolization to ensure utilization of angioembolization in an optimal sequence for patient survival.

## Background

Pelvic fracture management is one of the most complex treatment strategies [1]. Published guidelines offer varying approaches to care for hemodynamically unstable pelvic fractures [2–6]. The World Society of Emergency Surgeons (WSES) and Western Trauma Association (WTA) recommend selective angioembolization after pelvic packing [2, 3]. Eastern Association for the Surgery of Trauma (EAST) and Advanced Trauma Life Support (ATLS) suggest angioembolization after circumferential compression device application [5, 6]. Trauma Quality Improvement Program (TQIP) [4] utilizes angioembolization after external fixation and pelvic packing, or last when in extremis. There remains a high level of ambiguity on the optimal management of patients with hemodynamic unstable pelvic fractures across guidelines [2–6].

It is known that the time from presentation to angiography affects mortality in cases where angioembolization is needed [7]. Tanizaki et al. found a 4-fold increase in mortality rates for patients who went to angiography 60 min after arrival when compared to those who went within 60 min [7]. This is at least part of the reason that the American College of Surgeons (ACS) requires an interventional radiologist available within 30 min at Level I trauma centers [8]. Although, it has been reported that not all Level I trauma centers have IR on-site, the full extent of IR availability has not been described; therefore it is unclear if angiography within 1 h of arrival is possible [9].

## Methods

This anonymous cross-sectional survey of 158 trauma medical directors at United States ACS-verified Level I trauma centers was approved by the Western Institutional Review Board. The contact list was derived from the ACS website, individual trauma center’s websites, and via telephone. To view the invitation list, view the Appendix. Coauthors piloted the web-based survey prior to its online dissemination through SurveyMonkey Inc. (San Mateo, California; www.surveymonkey.com). Six invitations, that contained the approved partial waiver of consent, were emailed from March 1, 2018 to June 26, 2018. Participants were called to verify email receipt if they had not responded upon sending the final two invitations. No compensation was provided, and participation was voluntary. Trauma medical directors or an assigned colleague completed the survey and are referred to as “participants”.

The study hypotheses were 1) that IR was not on-site and prepared for intervention within 60 min, and 2) arrival times for IR when working off-site and the time for IR to prepare for intervention would be associated with the priority treatment sequence for angioembolization. The survey included 46 questions regarding IR availability and pelvic fracture management practices. To view questions pertaining to this paper, visit: http://bit.ly/SurveyIR. Irrelevant questions were skipped based on prior responses using SurveyMonkey’s ‘skip logic’, and participants could skip any question; therefore, there are missing responses for individual questions. Analysis was completed on SAS 9.4 (Cary, NC) software. Categorical data were summarized as counts and proportions. The median (interquartile range [IQR]) sequence for angioembolization was compared by both the arrival time for IR, and by the time for IR to prepare for intervention using the Kruskal-Wallis test. Ordinal logistic regression was used to determine if the arrival time for IR, or the time for IR to prepare for intervention was associated with the priority treatment sequence for angioembolization. All hypothesis tests were two-tailed with an alpha of 0.05.

## Results

The response rate was 25% (40/158). Of the survey responses, 90% (36/40) completed and 10% (4/40) partially completed the survey; all responses were included. Participating Level I trauma centers’ characteristics have been reported [10]. The median (IQR) survey completion time was 11 min (8, 21). No pelvic fracture protocol was implemented at 28% (11/40) of participating Level I trauma centers (Table 1). The most common pelvic fracture guideline followed was the EAST guideline (23% [11/40]). A majority of participants preferred using angioembolization before pelvic packing (63% [17/27]). Contrast extravasation was the most common angioembolization indicator (60% [21/35]).

**Table 1:** Angiography for Pelvic Fracture Management at Level I Trauma Centers

Questions and Possible Responses	% (n)	n
What agency’s guideline is your trauma center following for pelvic fracture management?		
No guideline in place	28% (11)	40
Eastern Association for the Surgery of Trauma	23% (9)	
Hospital developed protocol	18% (7)	
Western Trauma Association	15% (6)	
Trauma Quality Improvement Program	8% (3)	
Advanced Trauma Life Support	5% (2)	
Agency not specified	5% (2)	
Does your hospital use both angioembolization and pelvic packing for pelvic fracture management?		
Yes	85% (23)	27
No	15% (4)	
Angioembolization or Pelvic Packing First?		
Angioembolization	63% (17)	27
Pelvic packing	37% (10)	
Does your trauma center have a mobile c-arm?		
Yes	100% (36)	36
No	0	
Indicators for angioembolization		
Contrast extravasation	60% (21)	35^a^
Hemodynamically unstable	46% (16)	
Physician’s discretion	17% (6)	
Hemodynamically stable	14% (5)	
APC, LC, or VS fracture pattern	9% (3)	
After pelvic packing	9% (3)	
After a circumferential compression device	9% (3)	
Pelvic hematoma	9% (3)	
Requiring ongoing transfusions	9% (3)	
After REBOA	3% (1)	
Pseudoaneurysm	3% (1)	
When contrast extravasation is absent on computed tomography, but the patient is hemodynamically unstable, is angioembolization considered a treatment option?		
Yes	70% (25)	36
No	31% (11)	
What treatment is utilized while waiting for IR to set-up?		
Circumferential compression device	90% (35)	39^a^
Pelvic packing	64% (25)	
REBOA	44% (17)	
Exploratory laparotomy	31% (12)	
Other (massive transfusion protocol)	3% (1)	

^a^ Participants allowed to select multiple responses, *IR* interventional radiology, *REBOA* resuscitative endovascular balloon occlusion of the aorta, *APC* anterior-posterior compression, *LC* lateral compression, *VS* vertical shear

Fifty-four percent (20/37) of the represented Level I trauma centers had 24-h on-site IR coverage (Table 2). The remaining had on-call IR coverage; 13% (2/16) of participants reported IR was on-call for 24 h/day, and 31% (5/16) reported IR was on-call for 12 h/day. A majority (71% [12/17]) of participants reported a 21–30-min arrival time for IR when on-call. In addition to arrival times, 46% (17/37) of participants reported an IR procedure set-up time of 31–120 min. Most participants provided temporalizing stabilization through circumferential compression devices, pelvic packing, or REBOA while waiting for IR to prepare for intervention (Table 1).

**Table 2:** Interventional Radiology Coverage at Level I Trauma Centers

Questions and Responses	% (n)	n
Does the interventional radiology department have on-site coverage 24-h a day?		
Yes	54% (20)	37
No	46% (17)	
How many hours per day is there an interventional radiologist available by call only?		
8	13% (2)	16
10	19% (3)	
12	31% (5)	
13	6% (1)	
14	13% (2)	
15	6% (1)	
24	13% (2)	
Approximately how long does it take for an interventional radiologist to arrive when working off-site?		
0–10 min	0	17
11–20 min	6% (1)	
21–30 min	71% (12)	
≥ 31 min	24% (4)	
Approximately how long does it take for IR to set-up for angioembolization once an interventional radiologist is on-site?		
0–30 min	54% (20)	37
31–60 min	35% (13)	
61–120 min	11% (4)	
120–180 min	0	
> 180 min	0	

*IR* Interventional radiology

We previously reported the priority treatment sequence for hemodynamically unstable pelvic fractures [10]. The median priority treatment sequence for angioembolization was examined according to the IR arrival time when working off-site and to the time it took IR to prepare for intervention (Table 3). There was no significant relationship between the arrival times, or the intervention preparation time, and median priority sequence of angioembolization. The intervention preparation time, and the arrival time for IR when working off-site, were not significant predictors for the priority treatment sequence of angioembolization, (Table 4). This is evidenced by a lack of significance for these variables as well as a lack of significance in the Hosmer-Lemeshow goodness of fit p-value.

**Table 3:** Interventional Radiology Arrival and Preparation Times with the Median Treatment Sequence for Angioembolization

	Median (IQR)	n^a^/N^b^	*p*
Time for interventional radiologists to arrive			
0^c^	1 (1, 3)	8/20	0.84
0–10 min	N/A	0/0	
11–20 min	2 (2, 2)	0/1	
21–30 min	1 (1, 2)	5/12	
≥31 min	1.5 (1, 2)	1/4	
Time for interventional radiology to prepare for intervention			
0–30 min	1 (1, 2)	8/20	0.72
31–60 min	1 (1, 2)	5/13	
61–120 min	2 (1, 3)	1/4	

^a^ number of patients who chose to use angioembolization first, ^b^ total number of patients responding, ^c^ participants who indicated their interventional radiology department has on-site coverage 24-h a day

**Table 4:** Odds of Subsequent Priority Sequence of Angioembolization for IR Arrival and Preparation Times

	OR (CI)	*p*	H-L GOF
Time for interventional radiologists to arrive			
0^a^	Ref.	0.24	< 0.0001
0–10 min	N/A		
11–20 min	0.48 (0.06, 3.92)		
21–30 min	0.39 (0.15, 1.02)		
≥31 min	1.12 (0.27, 4.67)		
Time for interventional radiology to prepare for intervention			
0–30 min	Ref.	0.06	< 0.0001
31–60 min	0.32 (0.12, 0.84)		
61–120 min	0.90 (0.29, 2.75)		

*IR* interventional radiology, *OR* odds ratio, *CI* confidence interval, *H-L GOF* Hosmer-Lemmeshow goodness of fit, ^a^ participants who indicated their interventional radiology department has on-site coverage 24-h a day

## Discussion

This study surveyed 25% of ACS-verified Level I trauma centers on angiography practices and IR availability to treat hemodynamically unstable pelvic fractures. We failed to reject the null hypotheses; IR availability was variable across Level I trauma centers and did not significantly affect the priority treatment sequence of angioembolization. A majority of participants utilized angioembolization and pelvic packing, supporting the argument that pelvic packing and angioembolization should be complementary, not competitive, as the treatments target either venous or arterial hemorrhages [11]. Angioembolization primarily treats arterial bleeds, representing 10–20% of hemorrhaging, but cannot treat the majority of hemorrhaging from venous and cancellous sources [2]. Although the priority sequence for angioembolization and pelvic packing continues to be debated, this study observed a reported preference.

The majority of participants used angioembolization before pelvic packing. Contrary to this, it has been suggested that pelvic packing may be more efficient when used before angioembolization as it treats the majority of pelvic hemorrhaging [2]. Predicting the need for angioembolization has proven difficult; applying pelvic packing first allows for identification of the bleed source and determination of the need for angioembolization [3, 9, 11–13]. Additionally, several studies found a shorter time from admission to pelvic packing than angiography [13–16]. The use of angioembolization before pelvic packing may be due to EAST guideline, being the most commonly followed guideline, recommending angioembolization first [5]. Although Cothren et al. [17] stated preperitoneal pelvic packing can supplant angioembolization needs, this study found that most participants utilized angioembolization and prioritized it earlier than other treatment modalities.

It is our observation that a common reason for pelvic packing application is due to excessive wait times for IR. Despite the prevalence of angioembolization before pelvic packing, roughly half of the responding Level I trauma centers did not have 24-h on-site IR coverage. Furthermore, many participants reported arrival and IR procedure preparation times in excess of 30 min; some as long as 1–2 h. Ironically, this study revealed a lack of association between the amount of time it took IR to prepare for intervention and the priority treatment sequence of angioembolization for patients with hemodynamically unstable pelvic fractures. Yet, all participants reported utilization of alternative treatments while IR prepared for intervention. Not surprisingly, circumferential compression device was the most common treatment utilized while waiting; which is non-invasive and easily applied [2]. Pelvic packing was also a common treatment modality utilized while IR prepared; a sequence described by Burlew et al. [9] Almost half the participants indicated REBOA was utilized while IR prepared for intervention, suggesting more widespread use than previously reported [18]. The variety of treatment modalities used while waiting is no surprise, given that no guideline provides direction in this situation [2–6]. Therefore, more data is needed to determine the optimal priority treatment when IR is not prepared for intervention.

### Limitations

The response rate of 25% was a limitation as the participants responses may not be representative of all Level I trauma centers. The online-only survey format may have negatively impacted the response rate as some trauma medical directors noted a preference towards paper surveys. Some Level I trauma centers had outdated contact information for the trauma medical director which resulted in less email invitations being sent to the participant. Responses may have been subject to self-report and recall biases. Survey anonymity and instructions to have protocols on-hand were precautions to reduce these biases. In addition, mortality data was not collected; therefore we cannot conclude what practices were associated with better outcomes.

## Conclusions

The optimal priority treatment sequence for pelvic fractures has not been definitively determined. The reported IR arrival time and time to prepare for intervention did not significantly predict the priority treatment sequence of angioembolization; suggesting the priority treatment sequence was not altered based on these timing metrics. The use of angioembolization first may only be viable to prevent mortality at centers with 24-h on-site IR availability or faster preparation times. Level I trauma centers should review the literature and guidelines on time to angioembolization, their own arrival times for interventional radiology when working off-site, and their intervention preparation times for angioembolization to ensure utilization of the treatment options in an optimal sequence for patient survival.

## Data Availability

Data for this study is stored on Sharefile, an electronic HIPAA and HITECH-compliant platform that ensures all transmissions are fully encrypted, end-to-end. The datasets used for analysis for the current study are available from the corresponding author on reasonable request.

## Appendix

Level I Trauma Centers Invited to Participate in the Survey

**Table:**

Albany Medical Center	
Banner University Medical Center – Tucson	
Banner University Medical Center Phoenix	
Barnes-Jewish Hospital	
Baylor University Medical Center at Dallas	
Baystate Medical Center	
Beaumont Hospital - Royal Oak Campus	
Bellevue Hospital Center	
Ben Taub Hospital - Harris Health System	
Beth Israel Deaconess Medical Center	
Boston Medical Center	
Brigham and Women’s Hospital	
Bronson Methodist Hospital	
Brooke Army Medical Center	
Carilion Roanoke Memorial Hospital	
Carolinas Medical Center	
Cedars-Sinai Medical Center	
Charleston Area Medical Center	
Christiana Care Health System	
Cleveland Clinic Akron General	
Community Regional Medical Center	
Cooper University Health Care	
Dartmouth-Hitchcock Medical Center	
Dell Seton Medical Center at the University of Texas	
Denver Health Medical Center	
Detroit Receiving Hospital	
Dignity Health Chandler Regional Medical Center	
Dignity Health St. Joseph’s Hospital and Medical Center	
Duke University Hospital	
East Texas Medical Center Tyler	
Erie County Medical Center	
Eskenazi Health	
Froedtert Hospital	
George Washington University Hospital	
Grady Memorial Hospital	
Grant Medical Center	
Greenville Memorial Hospital	
Harbor UCLA Medical Center	
Hartford Hospital	
Hennepin County Medical Center	
Henry Ford Hospital	
Highland Hospital/A member of Alameda Health System	
HonorHealth John C. Lincoln Medical Center	
HonorHealth Scottsdale Osborn Medical Center	
Howard University Hospital	
Hurley Medical Center	
Indiana University Health Methodist Hospital	
Inova Fairfax Hospital	
Intermountain Medical Center	
Iowa Methodist Medical Center	
Jackson Memorial Hospital	
Jacobi Medical Center	
Jamaica Hospital Medical Center	
JPS Health Network	
Kendall Regional Medical Center	
LAC + USC Medical Center	
Legacy Emanuel Medical Center	
Lincoln Medical and Mental Health Center	
Loyola University Medical Center	
Maine Medical Center	
Maricopa Integrated Health System - Maricopa Medical Center	
Massachusetts General Hospital	
Mayo Clinic Rochester Trauma Centers	
Medical Center Navient Health	
Medical University of South Carolina	
MedStar Washington Hospital Center	
Memorial Hermann Hospital System – Houston	
Memorial Regional Hospital	
Mercy Health - St. Elizabeth Youngstown Hospital	
Mercy Health - St. Vincent Medical Center	
Methodist Dallas Medical Center	
MetroHealth Medical Center	
Miami Valley Hospital	
Morristown Medical Center	
Nassau University Medical Center	
Nebraska Medicine - Nebraska Medical Center	
New Jersey Trauma Center at the University Hospital	
New York Presbyterian Hospital - Weill Cornell Medical Center	
New York-Presbyterian – Queens	
North Memorial Health Hospital	
Northwell Health North Shore University Hospital	
Northwell Health Staten Island University Hospital	
NYC Health and Hospitals - Elmhurst	
NYC Health and Hospitals - Kings County	
NYU Langone Hospital – Brooklyn	
NYU Winthrop Hospital	
Oregon Health & Science University	
OU Medical Center	
Palmetto Health Richland	
Parkland Health & Hospital System	
Penrose Hospital	
ProMedica Toledo Hospital	
Regions Hospital	
Rhode Island Hospital	
Richmond University Medical Center	
Robert Wood Johnson University Hospital	
Ronald Reagan UCLA Medical Center	
Santa Barbara Cottage Hospital	
Santa Clara Valley Medical Center	
Scott & White Memorial Hospital – Temple	
Scripps Mercy Hospital	
Sparrow Hospital	
Spectrum Health - Butterworth Hospital	
SSM Health Saint Louis University Hospital	
St. Anthony Hospital	
St. Joseph Mercy Hospital - Ann Arbor	
St. Vincent Indianapolis Hospital	
Stanford Health Care	
Stony Brook Medicine	
Summa Akron City Hospital	
Swedish Medical Center	
Tampa General Hospital	
The Medical Center of Plano	
The Ohio State University Wexner Medical Center	
The Queen’s Medical Center	
The University of Kansas Hospital	
The University of Toledo Medical Center	
Tufts Medical Center	
UC Irvine Health	
UC San Diego Medical Center	
UMASS Memorial Medical Center	
University Health System - San Antonio	
University Health-Shreveport	
University Hospitals Cleveland Medical Center	
University Medical Center – Lubbock	
University Medical Center New Orleans	
University Medical Center of El Paso	
University Medical Center of Southern Nevada	
University of Alabama at Birmingham Hospital	
University of Arkansas for Medical Sciences	
University of California, Davis Medical Center	
University of Cincinnati Medical Center	
University of Iowa Hospitals & Clinics	
University of Kentucky Albert B. Chandler Hospital	
University of Louisville Hospital	
University of Michigan Health System	
University of Missouri Health System	
University of New Mexico Hospital	
University of North Carolina Hospital	
University of Rochester Medical Center/Strong Memorial Hospital	
University of Tennessee Medical Center	
University of Texas Medical Branch	
University of Utah Health Care	
University of Vermont Medical Center	
University of Virginia Health System	
University of Wisconsin Hospital and Clinics Authority	
Upstate University Hospital	
Vanderbilt University Medical Center	
Via Christi Hospitals – Wichita	
Vidant Medical Center	
Virginia Commonwealth University Medical Center	
Wake Forest Baptist Medical Center	
WakeMed Health & Hospitals	
Wesley Medical Center	
West Virginia University Hospitals-J.W. Ruby Memorial Hospital	
Westchester Medical Center	
Yale-New Haven Hospital	
Zuckerberg San Francisco General Hospital and Trauma Center	

## Abbreviations

ACS: American College of Surgeons
ATLS: Advanced Trauma Life Support
EAST: Eastern Association for the Surgery of Trauma
IR: Interventional Radiology
REBOA: Resuscitative Endovascular Balloon Occlusion of the Aorta
TQIP: Trauma Quality Improvement Program
WSES: World Society of Emergency Surgeons
WTA: Western Trauma Association
